# Tumor Budding and Poorly Differentiated Clusters in Small Intestinal Adenocarcinoma

**DOI:** 10.3390/cancers12082199

**Published:** 2020-08-06

**Authors:** Sun-Young Jun, Joon-Yong Chung, Nara Yoon, Eun Sun Jung, Young-Ha Oh, Seung-Mo Hong

**Affiliations:** 1 Department of Pathology, Incheon St. Mary’s Hospital, College of Medicine, The Catholic University of Korea, Seoul 21431, Korea; waxggul@gmail.com; 2 Laboratory of Pathology, Center for Cancer Research, National Cancer Institute, National Institutes of Health, Bethesda, MD 20892, USA; chungjo@mail.nih.gov; 3 Department of Pathology, Eunpyeong St. Mary’s Hospital, College of Medicine, The Catholic University of Korea, Seoul 03312, Korea; esjung@catholic.ac.kr; 4 Department of Pathology, Hanyang University College of Medicine, Guri 11923, Korea; yhoh@hanyang.ac.kr; 5 Department of Pathology, Asan Medical Center, University of Ulsan College of Medicine, Seoul 05505, Korea

**Keywords:** small intestine, adenocarcinoma, tumor budding, poorly differentiated clusters, prognosis

## Abstract

The clinicopathologic and prognostic significances of tumor budding (TB) and poorly-differentiated clusters (PDC) have not been investigated in small intestinal adenocarcinomas (SIACs). In 236 surgically-resected SIACs, we counted TB (single cells or clusters ≤4 tumor cells) and PDC (clusters ≥5 tumor cells) at the peritumoral-invasive front (p) and in the intratumoral area (i) independently to classify as grade-1 (≤4), grade-2 (5–9), or grade-3 (≥10). Consequently, grades-2 and -3 were considered high-grade. High-pTB, -iTB, -pPDC, and -iPDC were observed in 174 (73.7%), 129 (54.7%), 118 (50.0%), and 85 (36.0%) cases, respectively. High-TB/PDCs were more frequently observed in tumors with high-grade, higher T- and N-categories and stage grouping, and perineural or lymphovascular invasion. Patients with high-TB/PDC had a shorter survival than those with low-TB/PDC. In a multivariate analysis, high-pTB, nonintestinal type, high N-category, retroperitoneal seeding, and microsatellite-stable were worse independent-prognostic predictors. Subgroup analysis demonstrated that patients with high-pTB showed worse survival (median: 42.5 months) than those with low-pTB (133.7 months; *p* = 0.007) in the lower stage (stages I–II) group. High-TB/PDC, both in peritumoral and intratumoral localizations, were associated with aggressive behaviors in SIACs. High-pTB can be used as an adverse prognostic indicator in SIAC patients, especially when patients are in early disease stages.

## 1. Introduction

Small intestinal adenocarcinoma (SIAC) is very rare, compromising less than 3% of gastrointestinal (GI) malignancies [1]. In the United States, the overall incidence of SIAC has been increasing slowly since the early 1990s [2], and an estimated 11,110 new cases are expected to be diagnosed in 2020 [3]. In Korea, 894 new cases were diagnosed with SIAC in 2016 [4].

SIAC tends to be found at an advanced stage due to nonspecific symptoms and difficulty detecting the tumors [5]. Moreover, there are no appropriate screening programs, even for potentially high-risk individuals [6]. To make matters worse, few studies have been published to help guide the management of SIAC owing to the rarity of the tumors [6]. Although the National Comprehensive Cancer Network (NCCN) has recently published guidelines for the treatment of SIAC [7], management of SIAC has been historically extrapolated from colorectal cancer (CRC). Patient outcomes for SIAC are inferior to those for CRC at all stages of diagnosis [1,2].

Previous studies on SIACs have concentrated on comparisons with CRCs because of the anatomical proximity and histopathological similarities [5,8,9,10,11]. As a result, few prognostic factors have been identified in SIACs such as tumor location (duodenum vs. jejunum and ileum), microsatellite instability (MSI), tumor-infiltrating lymphocytes (TILs), and *KRAS* or *BRAF* mutations [9,10,11]. Kim et al. demonstrated that the complete epithelial–mesenchymal transition (EMT) phenotype, exhibiting an absence of E-cadherin expression and presence of vimentin and/or fibronectin expression, represented a poor clinical outcome in patients with SIAC [12].

Tumor budding (TB), both intratumoral (buds in the tumor center, iTB) and peritumoral (buds at the invasive front, pTB), has been well known to be a morphologic manifestation of EMT [13,14]. TB is defined as a single tumor cell or a small tumor cluster consisting of as many as four tumor cells [15]. Recently, the International Consensus Conference on Tumor Budding (ITBCC) recommended an evidence-based, standardized scoring system for TB to be used in CRC, which can be categorized into low (0–4 buds), intermediate (5–9 buds), and high (10 or more buds) levels based on counting buds on hematoxylin and eosin (H and E) slides in one hotspot of 20× objective lens [13]. Over the past decade, there was mounting evidence of the adverse effect of TB in CRCs and other GI malignancies, most of which revealed pTB as a strong predictor of lymphovascular invasion, tumor recurrence, nodal and distant metastases, and poor patients’ survival [13,16,17,18,19,20,21,22,23,24]. On the other hand, Lugli et al. found a strong relationship between pTB and iTB and emphasized their similar prognostic significance in CRCs [23].

Poorly-differentiated clusters (PDC) were first described in 2008 and similar to TB, they were a highly reproducible and relevant factor for predicting the prognosis and metastatic risk of patients with CRCs [25,26,27]. PDC, defined as solid cancer nests comprising five or more cancer cells and lacking glandular formation, have shared an identical grading system with TB since 2012 [15,28]. Ueno et al. proposed that PDC can predict prognostic outcome in CRC patients more accurately than TB [28]. Tumor grading via PDC quantification was shown to be reliable and more informative than tumor-node-metastasis (TNM) staging for predicting prognosis in CRC [29]. Bertoni et al. suggested that pPDC and iPDC are biologically different based on their discordant pattern of β-catenin and E-cadherin expression [30]. Meanwhile, high levels of PDC in tumors may be linked to RAS oncogenic mutations inducing dedifferentiation of the EMT process [31]. However, the clinicopathologic and prognostic significances of TB and PDC have not been investigated in SIACs.

In this study, we analyzed TB and PDC in both peritumoral and intratumoral regions in detail and verified their associations with clinicopathologic variables and prognostic values in SIAC.

## 2. Materials and Methods

### 2.1. Tissue Samples

A total of 236 surgically resected primary SIAC cases were enrolled, including 231 previously reported cases [9] and 4 additional cases. Primary duodenal, jejunal, and ileal carcinomas were included in this study. On the contrary, we excluded carcinomas extending from the ampulla of Vater into the small intestines. This study was approved by the Institutional Review Board of Incheon St. Mary’s Hospital (OC14OIMI0133), and patient consent was waived due to the retrospectively obtained and anonymized data of this study.

Clinicopathologic information collected as part of a previous study was updated [9]. Histologic subtypes were classified by the 5th edition of the World Health Organization (WHO) classification [1]. A tumor containing extracellular mucin greater than 50% of the tumor volume was designated as mucinous carcinoma [1]. A signet ring cell carcinoma was defined when more than 50% of the tumor cells had prominent intracytoplasmic mucin, typically with the displacement of the nuclei [15]. T- and N-categories and stage grouping were evaluated according to the 8th American Joint Committee on Cancer (AJCC) staging system [32]. In addition, we classified SIACs into intestinal and nonintestinal immune-phenotypes based on the combined CDX2 and MUC1 expression patterns, as previously reported [33]. SIAC cases expressing CDX2+/MUC1- were considered intestinal immune-phenotypes, while the other SIACs with CDX2-/MUC1+, CDX2+/MUC1+, and CDX2-/MUC1-immunoreactivities were categorized as nonintestinal [33]. *KRAS* and *BRAF* genotyping results and microsatellite instability (MSI) status obtained from the previous studies were also included [9,11]. Briefly, mutations in codons 12 and 13 of *KRAS* and codon 600 of *BRAF* exon 15 were analyzed by Sanger sequencing using formalin-fixed paraffin-embedded tissue blocks [11]. For MSI status assessment, five quasi-monomorphic mononucleotide repeats, including BAT25, BAT26, NR21, NR24, and NR27, were analyzed using a single multiplex polymerase chain reaction [9]. According to the National Cancer Institute (NCI) guidelines, tumors were divided into MSI-high (instability at ≥2 mononucleotide loci), MSI-low (instability at a single mononucleotide locus), and microsatellite stable (MSS; no instability at any of the loci tested) [9].

### 2.2. TB and PDC

**Figure 1:**
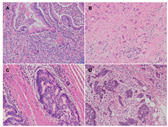
Representative images of tumor budding (TB) and poorly-differentiated clusters (PDC). (**A,B**) TB is defined as a single tumor cell or as a tumor cell cluster of as many as 4 cancer cells. In contrast, (**C,D**) a tumor cell cluster containing 5 or more cancer cells and lacking glandular formation is categorized as PDC. Based on the counting levels, they are independently categorized as (**A**) low TB, (**B**) high TB, (**C**) low PDC, and (**D**) high PDC (all, H and E, 200× magnification).

Representative H and E-stained, full-faced sections containing the deepest part of the invasive front of the tumor were selected. TB was defined as a single cell or clusters of as many as 4 tumor cells, whereas PDC was defined as clusters of 5 or more tumor cells without glandular formation (Figure 1A–D) [15]. TB and PDC were independently counted at the peritumoral invasive front (p) and in the intratumoral area (i) by two experienced GI pathologists (S.-Y.J. and N.Y.) blinded to clinicopathologic information using an Olympus BX-53 microscope (Olympus, Tokyo, Japan). Discrepancies were resolved by simultaneous reevaluation and discussion. At least 10 individual fields at medium power (10× objective) were scanned to identify the “hotspot” of TB and PDC, and TB and PDC were counted in one hotspot of 20× objective (area, 0.785 mm^2^) to be categorized into grade-1 (0 to 4), grade-2 (5 to 9), or grade-3 (≥10), according to the ITBCC criteria [13]. Consequently, grade-2 or -3 were considered high levels of TB and PDC, independently (Figure 1).

### 2.3. Statistical Analysis

All statistical analyses were performed using SPSS software (version 17.0; SPSS Inc., Chicago, IL, USA). Mean values were compared by the Student’s t test or simple analysis of variance. Associations between clinicopathologic factors were assessed using χ^2^ and Fisher’s exact test. Logistic regression models were used to evaluate multivariate associations. Overall survival probabilities were plotted with the Kaplan–Meier method, and the significance of differences in survival probabilities were probed using a log-rank test. Univariate and multivariate survival analyses were performed using the Cox proportional hazards modeling. A *p* value of < 0.05 was considered statistically significant.

## 3. Results

### 3.1. Clinicopathologic Features

The mean age of the SIAC patients was 59.6 ± 12.7 years, and the male to female ratio was 1.8. Tumor size was evaluated in 234 cases, and the mean tumor size was 4.3 ± 0.2 cm. The median follow-up period after surgical resection was 25.3 months (range, 0.3–168.4 months). SIACs were located in the duodenum in 142 cases (60.2%), the jejunum in 61 (25.8%), and the ileum in 33 (14.0%). According to the AJCC staging scheme, 5 tumors were categorized as Tis (2.1%), 14 as T1 (5.9%), 14 as T2 (5.9%), 70 as T3 (29.7%), and 133 as T4 (56.4%). Lymph nodes were examined in 219 cases, and half of them were N0 (111 cases, 50.7%). Nodal metastases were seen in 108 cases, including 55 N1 (25.1%) and 53 N2 (24.2%). Consequently, the tumors were classified into stages 0 (5 cases, 2.3%), I (23, 10.5%), IIA (33, 15.1%), IIB (50, 22.8%), IIIA (55, 25.1%), and IIIB (53, 24.2%). The combined expression patterns of CDX2 and MUC1 were assessed in 230 interpretable tumors [33]. Among 230 SIACs, 153 (66.5%) cases were classified as nonintestinal and 77 (33.5%) were intestinal. The mutation status of *KRAS* and *BRAF* was evaluated in 190 and 178 patients, respectively, as previously described [9]. *KRAS* and *BRAF* mutations were identified in 32.1% (61/190) and 1.1% (2/178) of cases, respectively [11]. MSI analysis was available in 230 SIAC cases. MSI was found in 50 cases (21.7%), all of which were MSI-high [9].

pTB was variably observed in 88.1% (208/236), of which levels were up to 60 (mean, 11.4 ± 11.0). iTB was identified in 78.0% (184/236), ranging up to 600 (mean, 12.9 ± 6.0). pPDC and iPDC were seen in 86.4% (204/236; maximum, 36; mean, 5.9 ± 5.3) and 77.1% (182/236; maximum, 30; mean, 4.6 ± 5.1), respectively. Based on the counting level, two thirds of the tumors (155/236, 65.7%) were categorized as grade-3 of pTB. Meanwhile, 62 cases were grade-1 (26.3%) and 19 were as grade-2 (8.0%) of pTB. In iTB, half of the tumors were grade-1 (107 cases, 45.3%), and grades-2 and -3 were seen in 32 (13.6%) and 97 (41.1%) cases, respectively. In pPDC, 118 (50.0%) cases of the tumors were grade-1, 45 (19.1%) were grade-2, and 73 (30.9%) were grade-3. iPDC was classified into grade-1 in 151 (64.0%) cases, grade-2 in 34 (14.4%), and grade-3 in 51 (21.6%). After merging cases with grades-2 and -3 and categorizing them as high level, high pTB, iTB, pPDC, and iPDC were observed in 174 (73.7%), 129 (54.7%), 118 (50.0%), and 85 (36.0%) cases, respectively.

### 3.2. Associations between Clinicopathologic Factors and TB and PDC

The associations between clinicopathologic factors and TB and PDC are summarized in Table 1 and Table 2, respectively. In TB, high levels of both pTB and iTB were associated with aggressive behaviors of SIACs, including nodular or infiltrative growth pattern, high tumor grade, and perineural or lymphovascular invasion (*p* < 0.001, all), pancreatic invasion (*p* = 0.007 and *p* = 0.022, respectively), nodal metastasis (*p* < 0.001 and *p* = 0.002, respectively), and higher T- (*p* < 0.001, both) and N-categories (*p* < 0.001 and *p* = 0.006, respectively) and stage grouping (*p* < 0.001 and *p* = 0.006, respectively). Nonintestinal types of SIACs more frequently exhibited high levels of pTB and iTB (*p* < 0.001, both). On the other hand, high pTB was more commonly observed in patients with radiotherapy (*p* = 0.015), whereas high iTB was in signet ring cell carcinoma and undifferentiated carcinoma (*p* = 0.020) and in tumors with retroperitoneal seeding (*p* = 0.042).

In PDC, high levels of both pPDC and iPDC were also related to aggressiveness of SIACs: nodular or infiltrative growth pattern (*p* < 0.001 and *p* = 0.002, respectively), high tumor grade (*p* = 0.002 and *p* < 0.001, respectively), nodal metastasis (*p* < 0.001 and *p* = 0.001, respectively), higher T- (*p* < 0.001 and *p* = 0.002, respectively) and N-categories (*p* < 0.001 and *p* = 0.003, respectively) and stage grouping (*p* < 0.001 and *p* = 0.002, respectively), and perineural (*p* = 0.008 and *p* = 0.003, respectively) or lymphovascular (*p* = 0.004 and *p* < 0.001, respectively) invasion. In addition, all PDC were observed in high levels in patients with nonintestinal type SIACs (*p* = 0.029 in pPDC; *p* = 0.018 in iPDC) and radiotherapy (*p* = 0.047 in pPDC; *p* = 0.025 in iPDC). Meanwhile, SIACs with high pPDC more frequently had pancreatic invasion (*p* = 0.022), while those with high iPDC had retroperitoneal seeding (*p* = 0.046). In addition, pPDC was higher in older patients (*p* = 0.009). *KRAS* and *BRAF* mutations and MSI status were not associated with TB and PDC in SIAC.

### 3.3. Associations between pTB, iTB, pPDC, and iPDC

To focus our investigations on pTB, we assessed its correlation with iTB, pPDC, and iPDC (Table 3). In a multivariate logistic regression analysis, high pTB remained significantly more frequent in SIACs with high levels of iTB (odds ratio, 69.203; 95% confidence interval, 15.586–307.264) and pPDC (odds ratio, 17.294; 95% confidence interval, 5.932–50.420) (*p* < 0.001, both). There was no relationship between pTB and iPDC (*p* = 0.291).

### 3.4. Survival Analysis

**Figure 2:**
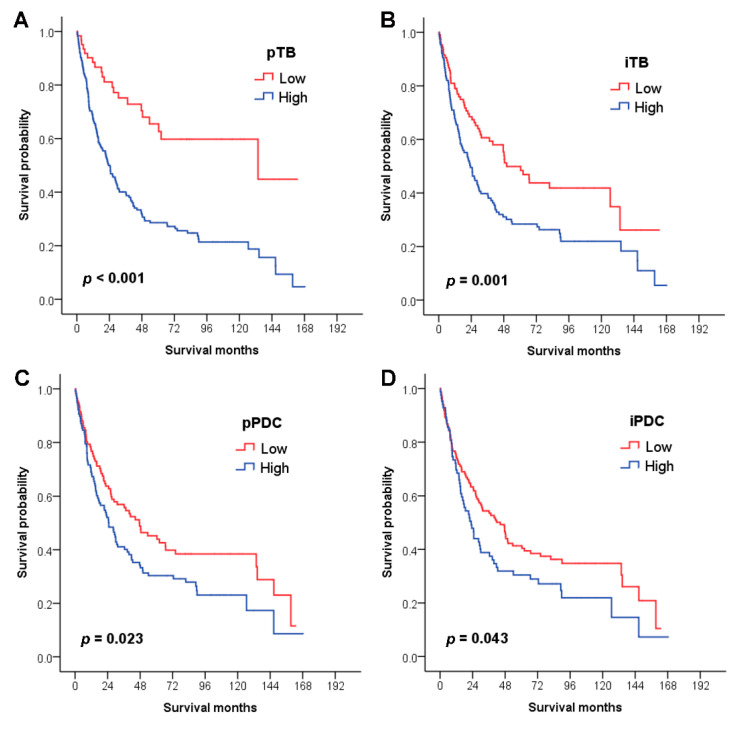
Survival analysis based on the status of TB and PDC. (**A**) SIAC patients with high pTB (median: 23.3 months) demonstrate significantly worse overall survival than those with low pTB (133.7 months; *p* < 0.001). (**B**) SIAC patients with high iTB (23.3 months) also have a shorter survival time than those with low iTB (50.1 months; *p* = 0.001). (**C**) SIAC patients with a high pPDC level (24.9 months) demonstrate significantly worse overall survival than those with a low level (47.4 months; *p* = 0.023). In addition, (**D**) SIAC patients with high iPDC (23.0 months) have worse survival than those with low iPDC (44.4 months; *p* = 0.043).

The results of the survival analyses of TB and PDC are described in Figure 2. Patients with high pTB (median: 23.3 months) demonstrated significantly worse overall survival than those with low pTB (133.7 months; *p* < 0.001, Figure 2A). Patients with high iTB (23.3 months) also had a shorter survival time than those with low iTB (50.1 months; *p* = 0.001, Figure 2B). In pPDC, a high level (24.9 months) was significantly associated with worse overall survival of patients than a low level (47.4 months; *p* = 0.023, Figure 2C). In addition, SIAC patients with high iPDC (23.0 months) showed worse survival than those with low iPDC (44.4 months; *p* = 0.043, Figure 2D). The following clinicopathologic factors were also associated with worse SIAC patient survival by univariate analysis (Table 4): nonintestinal phenotype (*p* < 0.001), distal location (*p* = 0.004), nodal metastasis (*p* < 0.001), higher T- and N-categories (*p* = 0.002 and *p* < 0.001, respectively) and stage groupings (*p* < 0.001), presence of retroperitoneal seeding (*p* < 0.001) and perineural and lymphovascular invasions (*p* = 0.004 and *p* < 0.001, respectively), radiotherapy (*p* = 0.008), and MSS (*p* = 0.029).

Multivariate analysis revealed that high pTB (*p* = 0.024), nonintestinal type (*p* = 0.001), high N-category (*p* = 0.001), retroperitoneal seeding (*p* = 0.039), and MSS (*p* = 0.003) were worse independent prognostic predictors (Table 4).

### 3.5. Survival Analysis of pTB based on Stage Groups

**Figure 3:**
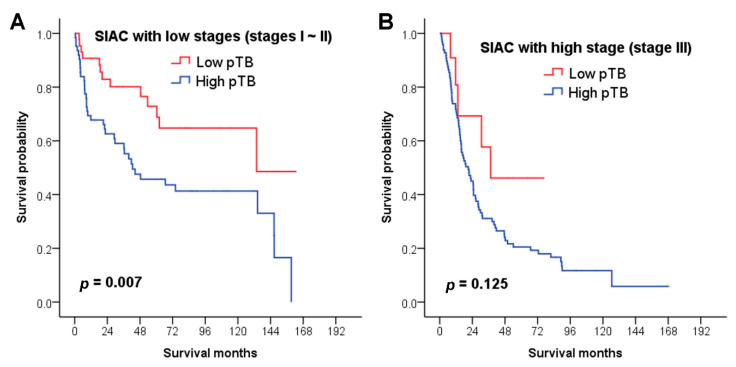
Survival analysis of pTB based on stage group. (**A**) In the lower stage (stages I–II) group (*n* = 106), the overall survival times for SIAC patients with high pTB (*n* = 63; median: 42.5 months) is significantly shorter than those with low pTB (*n* = 43; 133.7 months; *p* = 0.007). However, (**B**) in the higher stage group (*n* = 108), there is no statistical significance in survival time distribution between the patients with high pTB (*n* = 97; 21.0 months) and those with low pTB (*n* = 11; 37.4 months; *p* = 0.125).

The independent prognostic significance of pTB in SIACs, which was considered significant by multivariate analysis, was further evaluated according to stage (Figure 3). In the lower stage (stages I–II) group (*n* = 106), the overall survival times for SIAC patients with high pTB (*n* = 63; median: 42.5 months) were significantly shorter than those with low pTB (*n* = 43; 133.7 months; *p* = 0.007, Figure 3A). However, in the higher stage group (*n* = 108), there was no statistical significance in survival time distribution between the patients with high pTB (*n* = 97; 21.0 months) and those with low pTB (*n* = 11; 37.4 months; *p* = 0.125, Figure 3B).

## 4. Discussion

To the best of our knowledge, our study is the first to report the clinicopathologic significance of TB and PDC and their prognostic values in SIACs. In CRC, pTB and iTB have been well-known indicators linked to higher TNM stage and tumor grade, lymphovascular invasion, or nodal and distant metastases [13,22,24]. Furthermore, the similarities in adverse prognostic roles of pTB and iTB were described in a study by Lugli and colleagues [23]. In the present study, we observed that high levels of pTB and iTB were associated with aggressiveness of SIACs such as high tumor grade, higher T- and N-categories and stage grouping, nodular or infiltrative growth pattern, perineural or lymphovascular invasion, and pancreatic invasion. Moreover, SIAC patients with high pTB and/or iTB had significantly shorter overall survival. These findings are concordant with those of previous studies in CRC. However, the clinical impact of iTB was somewhat different from pTB. iTB seemed to qualify for investigation in the prospective setting [24]; for example, high iTB rate in the preoperative biopsy of CRC patients could be predictive of nodal and distant metastases [24]. Therefore, in endoscopically resected pT1 CRC, iTB could help to select the patients who required surgical resection due to a high risk of nodal and distant metastasis [13,34]. In addition, iTB in preoperative biopsies could identify CRC patients who may be eligible for neoadjuvant chemoradiotherapy and potentially predict tumor regression [13,35]. In subgroups of nodal negative CRC, iTB could also select the patients who might show aggressive behavior and benefit from adjuvant therapy [22]. Our findings suggest that iPDC seemed to have a similar biological significance to iTB, since iPDC in the preoperative biopsy could predict the metastatic risk of patients [25] as well as tumor response and clinical outcome in CRC treated with neoadjuvant chemoradiotherapy [36]. Unfortunately, we could not include the preoperative biopsy specimens and the detailed information about chemotherapy in this study owing to the limitation of a multi-institutional study. Therefore, to analyze the utility of iTB and/or iPDC in biopsy specimens for the therapeutic response and prognosis of patients with SIAC, further investigations that include patients treated with modern treatment strategies after biopsy are necessary.

We graded PDC according to a proposal by Ueno et al., which was identical to the TB grading system [28]. In the previous CRC study by Ueno and colleagues, pPDC was a prognostic indicator with more accuracy than pTB [28]. However, the prognostic effect of pTB might be obscured due to selection bias because the T1 category was excluded [28]. In the present study, we assessed the prognostic significances of pTB, iTB, pPDC, and iPDC independently by multivariate analysis as all of them were related to the survival of patients with SIAC. Among them, we found that pTB provided a significant predictive value, whereas the others did not (iTB, *p* = 0.675; pPDC, *p* = 0.932; iPDC, *p* = 0.291.). In addition, we identified a prognostic value of pTB for survival in the lower stage (stages I–II) group of SIAC. Concordant with our results, Prall et al. found an adverse prognostic effect of pTB, specifically in a series of stage I and II CRC patients (*n* = 186) [37]. Therefore, pTB status could be used as an additional prognostic predictor in surgically resected SIAC patients with lower disease stage. Moreover, we observed that SIAC patients with high pTB had worse overall survival than those with low pTB in the stage III group, but it was not statistically significant. Stage IV was not included in this study; thus, further studies with larger numbers of cases with stage IV disease are required to support the use of pTB as an adverse prognosticator even in high stages.

Both TB and PDC are understood as EMT phenomena, activated by the Wnt/β-catenin signaling pathway [38]. TB acquires a mesenchymal phenotype with nuclear translocation of β-catenin and concomitant loss of membranous E-cadherin expression resulting in the activation of the Wnt pathway [13,18]. In PDC, the L1 cell adhesion molecule (*L1CAM*), which generates epithelial cell migration as one of the target factors of the Wnt signaling pathway, was upregulated [38,39]. In addition, oncogenic *RAS* mutation was associated with high PDC in CRCs [31,40]. In the present study, we could not find any significant association between *KRAS* mutation with TB and PDC in SIACs. To evaluate the association with TB and/or PDC and immune-phenotypes of SIACs, we compared our results of TB and PDC with that of our previous study on CDX2 and MUC1 immunolabeling [33]. As is known, CDX2 expression is related to the intestinal phenotype, while MUC1 is mainly expressed in the pancreatic duct and superficial foveolar epithelium [33]. As a result, we could identify that the loss of intestinal differentiation exhibiting CDX2- and/or MUC1+ was related to high levels of TB and PDC in SIACs. Moreover, the nonintestinal immuno-phenotype was also revealed as an independent worse prognostic parameter. Therefore, it might be useful to examine CDX2 and MUC1 immunolabeling patterns as intestinal versus nonintestinal in biopsy specimens to predict the prognosis of a patient with SIAC since pTB cannot be evaluated in biopsy specimens without evaluating the invasion front.

Cancer cells can invade surrounding structures as cell clusters as well as through single-cell invasion [41]. In a CRC study using Ezrin immunolabeling, PDC could serve as a platform for converting the migration manner from collective-cell to single-cell invasion [42]. Three-dimensional reconstruction of 4 μm serially sectioned immunohistochemical staining slides of solid cancer, including CRCs, revealed that TB was connected with the main tumor mass [43]. Similarly, TB in isolation in one plane of a section could be traced to the neighboring neoplasm at the invasion front by electron microscopy and immunohistochemical staining [44]. These findings may represent sequential steps in tumor mass outgrowth via PDC to TB. TB may evolve from PDC by acquiring proliferative and aggregative activities [45].

The present study has some limitations. We selected only a single slide containing the deepest invasion front for evaluating TB and PDC, although all H and E slides were initially assessed to select a representative slide. However, a previous study demonstrated an excellent agreement between the multi-slide and single-slide methods of analysis for TB and PDC in CRCs using a Japanese (*n* = 283) and a Scottish (*n* = 163) cohort. Herein, the analysis from a single representative H and E slide evaluation containing the deepest invasion front was an efficient way of evaluating TB and PDC [46].

## 5. Conclusions

High levels of TB and PDC, both in peritumoral and intratumoral regions, were associated with aggressive behavior and poor survival outcomes of patients with SIAC. Among TB and PDC, pTB can be used as a powerful independent adverse prognostic indicator in patients with SIACs, especially when patients are in early disease stages (stage I or II).

## Figures and Tables

**Table 1:** Association between Clinicopathologic Factors and TB Status in SIAC

Variable		pTB, *N* (%)			iTB, *N* (%)		
		Low	High	*p*	Low	High	*p*
No. of patients		62 (26)	174 (74)		107 (45)	129 (55)	
Age (mean ± SD, years)		57.7 ± 14.2	60.3 ± 12.3	0.156	58.6 ± 13.0	60.5 ± 12.7	0.247
Size (mean ± SD, cm) ^a^		4.5 ± 2.5	4.3 ± 2.5	0.616	4.5 ± 2.4	4.2 ± 2.6	0.469
Age (years)	≤50	19 (34)	36 (66)	0.111	26 (47)	29 (53)	0.742
	>50	43 (24)	138 (76)		81 (45)	100 (55)	
Sex	Male	40 (26)	112 (74)	0.983	63 (41)	89 (59)	0.106
	Female	22 (26)	62 (74)		44 (52)	40 (48)	
Growth pattern ^a^	Polypoid	27 (61)	17 (39)	**<0.001**	35 (80)	9 (20)	**<0.001**
	Nodular	4 (23)	13 (77)		7 (41)	10 (59)	
	Infiltrative	28 (17)	139 (83)		59 (35)	108 (65)	
Location	Proximal (duodenum)	43 (30)	99 (70)	0.085	69 (49)	73 (51)	0.217
	Distal (jejunum, ileum)	19 (20)	75 (80)		38 (40)	56 (60)	
Histologic subtype	Tubular	58 (28)	152 (72)	0.256	96 (46)	114 (54)	**0.020**
	Mucinous	4 (36)	7 (64)		8 (73)	3 (27)	
	Signet ring cell	0	4 (100)		0	4 (100)	
	Undifferentiated	0	5 (100)		0	5 (100)	
	Medullary	0	6 (100)		3 (50)	3 (50)	
Immunophenotype ^a^	Intestinal	34 (44)	43 (56)	**<0.001**	51 (66)	26 (34)	**<0.001**
	Nonintestinal	26 (17)	127 (83)		54 (35)	99 (65)	
Differentiation	Low (Well to moderately)	59 (32)	128 (68)	**<0.001**	100 (54)	87 (46)	**<0.001**
	High (Poorly or undifferentiated)	3 (6)	46 (94)		7 (14)	42 (86)	
Pancreas invasion	Absent	48 (32)	101 (68)	**0.007**	76 (51)	73 (49)	**0.022**
	Present	14 (16)	73 (84)		31 (36)	56 (64)	
Other loop invasion	Absent	62 (27)	168 (73)	0.345	106 (46)	124 (54)	0.225
	Present	0	6 (100)		1 (17)	5 (83)	
Retroperitoneal seeding	Absent	61 (28)	160 (72)	0.125	104 (47)	117 (53)	**0.042**
	Present	1 (7)	14 (93)		3 (20)	12 (80)	
Lymphovascular invasion	Absent	52 (43)	68 (57)	**<0.001**	72 (60)	48 (40)	**<0.001**
	Present	10 (9)	106 (91)		35 (30)	81 (70)	
Perineural invasion	Absent	55 (35)	104 (65)	**<0.001**	87 (55)	72 (45)	**<0.001**
	Present	7 (9)	70 (91)		20 (26)	57 (74)	
Resection margin status ^a^	No involvement	54 (25)	159 (75)	0.058	98 (46)	115 (54)	1.000
	Cancer involvement	5 (56)	4 (44)		4 (44)	5 (56)	
Nodal metastasis ^a^	No	48 (43)	63 (57)	**<0.001**	62 (56)	49 (44)	**0.002**
	Yes	11 (10)	97 (90)		38 (35)	70 (65)	
T category ^b^	Low (T1–T2)	21 (75)	7 (25)	**<0.001**	25 (89)	3 (11)	**<0.001**
	High (T3–T4)	36 (18)	167 (82)		77 (38)	126 (62)	
N category ^a^	N0	48 (43)	63 (57)	**<0.001**	62 (56)	49 (44)	**0.006**
	N1	4 (7)	51 (93)		17 (31)	38 (69)	
	N2	7 (13)	46 (87)		21 (40)	32 (60)	
Stage grouping ^a,b^	Low (Stages I–II)	43 (41)	63 (59)	**<0.001**	57 (54)	49 (46)	**0.006**
	High (Stage III)	11 (10)	97 (90)		38 (35)	70 (65)	
Chemotherapy ^a^	No	46 (31)	103 (69)	0.062	70 (47)	79 (53)	0.530
	Yes	16 (19)	66 (81)		35 (43)	47 (57)	
Radiation therapy ^a^	No	60 (30)	143 (70)	**0.015**	95 (47)	108 (53)	0.339
	Yes	2 (7)	25 (93)		10 (37)	17 (63)	
*KRAS* genotype ^a^	Wild type	35 (27)	94 (73)	0.172	57 (44)	72 (56)	0.839
	Mutated	11 (18)	50 (82)		26 (43)	35 (57)	
*BRAF* genotype ^a^	Wild type	44 (25)	132 (75)	1.000	80 (45)	96 (55)	0.502
	Mutated	0	2 (100)		0	2 (100)	
MSI status ^a^	MSS	46 (25)	139 (75)	0.462	81 (44)	104 (56)	0.433
	MSI-high	15 (30)	35 (70)		25 (50)	25 (50)	

Small intestinal adenocarcinoma (SIAC); standard deviation (SD); microsatellite stable (MSS); microsatellite instability (MSI); significant *p*-values in bold; ^a^ calculated using only patients with adequate data; ^b^ excluded patients with Tis and stage 0.

**Table 2:** Association between Clinicopathologic Factors and PDC Status in SIAC

Variable		pPDC, *N* (%)			iPDC, *N* (%)		
		Low	High	*p*	Low	High	*p*
No. of patients		118 (50)	118 (50)		151 (64)	85 (36)	
Age (mean ± SD, years)		58.2 ± 14.1	61.1 ± 11.4	0.082	59.3 ± 13.3	60.2 ± 12.0	0.599
Size (mean ± SD, cm) ^a^		4.4 ± 2.5	4.3 ± 2.5	0.712	4.2 ± 2.3	4.5 ± 2.9	0.492
Age (years)	≤50	36 (66)	19 (34)	**0.009**	37 (67)	18 (33)	0.562
	>50	82 (45)	99 (55)		114 (63)	67 (37)	
Sex	Male	79 (52)	73 (48)	0.415	97 (64)	55 (36)	0.943
	Female	39 (46)	45 (54)		54 (64)	30 (36)	
Growth pattern ^a^	Polypoid	34 (77)	10 (23)	**<0.001**	38 (86)	6 (14)	**0.002**
	Nodular	9 (53)	8 (47)		11 (65)	6 (35)	
	Infiltrative	73 (44)	94 (56)		96 (58)	71 (42)	
Location	Proximal (duodenum)	73 (51)	69 (49)	0.595	95 (67)	47 (33)	0.251
	Distal (jejunum, ileum)	45 (48)	49 (52)		56 (60)	38 (40)	
Histologic subtype	Tubular	108 (51)	102 (49)	0.134	134 (64)	76 (36)	0.069
	Mucinous	5 (45)	6 (55)		8 (73)	3 (27)	
	Signet ring cell	2 (50)	2 (50)		4 (100)	0	
	Undifferentiated	3 (60)	2 (40)		4 (80)	1 (20)	
	Medullary	0	6 (100)		1 (17)	5 (83)	
Immunophenotype ^a^	Intestinal	46 (60)	31 (40)	**0.029**	57 (74)	20 (26)	**0.018**
	Nonintestinal	68 (44)	85 (56)		89 (58)	64 (42)	
Differentiation	Low (Well to moderately)	103 (55)	84 (45)	**0.002**	132 (71)	55 (29)	**<0.001**
	High (Poorly or undifferentiated)	15 (31)	34 (69)		19 (39)	30 (61)	
Pancreas invasion	Absent	83 (56)	66 (44)	**0.022**	101 (68)	48 (32)	0.111
	Present	35 (40)	52 (60)		50 (58)	37 (42)	
Other loop invasion	Absent	115 (50)	115 (50)	1.000	149 (65)	81 (35)	0.192
	Present	3 (50)	3 (50)		2 (33)	4 (67)	
Retroperitoneal seeding	Absent	110 (50)	111 (50)	0.790	145 (66)	76 (34)	**0.046**
	Present	8 (53)	7 (47)		6 (40)	9 (60)	
Lymphovascular invasion	Absent	71 (59)	49 (41)	**0.004**	93 (78)	27 (32)	**<0.001**
	Present	47 (40)	69 (60)		58 (50)	58 (50)	
Perineural invasion	Absent	89 (56)	70 (44)	**0.008**	112 (70)	47 (30)	**0.003**
	Present	29 (38)	48 (62)		39 (51)	38 (49)	
Resection margin status ^a^	No involvement	107 (50)	106 (50)	1.000	136 (64)	77 (36)	0.727
	Cancer involvement	4 (44)	5 (56)		5 (56)	4 (44)	
Nodal metastasis ^a^	No	73 (66)	38 (34)	**<0.001**	84 (76)	27 (24)	**0.001**
	Yes	40 (37)	68 (63)		58 (54)	50 (46)	
T category ^b^	Low (T1–T2)	26 (93)	2 (7)	**<0.001**	25 (89)	3 (11)	**0.002**
	High (T3–T4)	87 (43)	116 (57)		121 (60)	82 (40)	
N category ^a^	N0	73 (66)	38 (34)	**<0.001**	84 (76)	27 (24)	**0.003**
	N1	18 (33)	37 (67)		28 (51)	27 (49)	
	N2	22 (41)	31 (59)		30 (57)	23 (43)	
Stage grouping ^a,b^	Low (Stages I–II)	68 (64)	38 (36)	**<0.001**	79 (75)	27 (25)	**0.002**
	High (Stage III)	40 (37)	68 (63)		58 (54)	50 (46)	
Chemotherapy ^a^	No	82 (55)	67 (45)	0.105	100 (67)	49 (33)	0.139
	Yes	36 (44)	46 (56)		47 (57)	35 (43)	
Radiation therapy ^a^	No	109 (54)	94 (46)	**0.047**	135 (67)	68 (33)	**0.025**
	Yes	9 (33)	18 (67)		12 (44)	15 (56)	
*KRAS* genotype ^a^	Wild type	59 (46)	70 (54)	0.282	80 (62)	49 (38)	0.254
	Mutated	33 (54)	28 (46)		43 (71)	18 (29)	
*BRAF* genotype ^a^	Wild type	85 (48)	91 (52)	1.000	115 (65)	61 (35)	1.000
	Mutated	1 (50)	1 (50)		1 (50)	1 (50)	
MSI status ^a^	MSS	98 (53)	87 (47)	0.060	121 (65)	64 (35)	0.334
	MSI-high	19 (38)	31 (62)		29 (58)	21 (42)	

Significant *p*-values in bold; ^a^ calculated using only patients with adequate data; ^b^ excluded patients with Tis and stage 0.

**Table 3:** Association between pTB Status and iTB, pPDC, and iPDC in SIAC

Variable		Frequency Analysis, *N* (%)			Multivariate Logistic Regression Analysis	
		Low pTB	High pTB	*p*	OR (95% CI)	*p*
iTB	Low	60 (56)	47 (44)	**<0.001**	69.203 (15.586–307.264)	**<0.001**
	High	2 (2)	127 (98)			
pPDC	Low	57 (48)	61 (52)	**<0.001**	17.294 (5.932–50.420)	**<0.001**
	High	5 (4)	113 (96)			
iPDC	Low	59 (39)	92 (61)	**<0.001**	2.359 (0.479–11.609)	0.291
	High	3 (3)	82 (97)			

TB at the invasive front (pTB); TB in the tumor center (iTB); PDC at the invasive front (pPDC); PDC in the tumor center (iPDC); odds ratio (OR); confidence interval (CI); significant *p*-values in bold.

**Table 4:** Univariate and Multivariate Analyses of SIAC Patients.

Variable		Univariate			Multivariate	
		Median Survival (mo)	HR (95% CI)	*p*	HR (95% CI)	*p*
pTB	Low	133.7				
	High	23.3	2.947 (1.858–4.675)	**<0.001**	1.890 (1.086–3.289)	**0.024**
iTB	Low	50.1				
	High	23.3	1.711 (1.226–2.386)	**0.001**		
pPDC	Low	47.4				
	High	24.9	1.450 (1.051–2.001)	**0.023**		
iPDC	Low	44.4				
	High	23.0	1.398 (1.010–1.935)	**0.043**		
Location	Proximal	41.7				
	Distal	22.5	1.598 (1.157–2.206)	**0.004**	1.455 (0.992–2.134)	0.055
Immunophenotype ^a^	Intestinal	134.4				
	Nonintestinal	22.0	2.533 (1.701–3.773)	**<0.001**	1.999 (1.309–3.053)	**0.001**
Retroperitoneal seeding	Absent	37.4				
	Present	14.0	2.997 (1.714–5.242)	**<0.001**	2.079 (1.037–4.166)	**0.039**
Lymphovascular invasion	Absent	66.6				
	Present	17.8	2.357 (1.695–3.277)	**<0.001**	1.476 (0.971–2.242)	0.068
Perineural invasion	Absent	47.6				
	Present	17.8	1.635 (1.172–2.281)	**0.004**	1.051 (0.707–1.562)	0.805
Nodal metastasis ^a^	Absent	133.7				
	Present	21.6	2.401 (1.690–3.411)	**<0.001**		
T category ^b^	Low (T1–T2)	– ^c^				
	High (T3–T4)	26.3	3.167 (1.553–6.458)	**0.002**	1.128 (0.484–2.628)	0.780
N category ^a^				**<0.001**		**0.001**
	N0	133.7			1	-
	N1	28.4	1.987 (1.312–3.008)	**0.001**	1.445 (0.903–2.313)	0.125
	N2	17.3	2.981 (1.981–4.486)	**<0.001**	2.501 (1.549–4.036)	**<0.001**
Stage grouping ^a,b^	Low (Stages I–II)	133.7				
	High (Stage III)	21.6	2.342 (1.645–3.335)	**<0.001**		
Radiation therapy ^a^	No	38.5				
	Yes	23.3	1.824 (1.172–2.839)	**0.008**	0.935 (0.572–1.528)	0.789
MSI status ^a^	MSS	28.2				
	MSI-high	72.6	0.629 (0.414–0.954)	**0.029**	0.470 (0.287–0.769)	**0.003**

Hazard ratio (HR); confidence interval (CI); significant *p*-values in bold; ^a^ calculated using only patients with adequate data; ^b^ excluded patients with Tis and stage 0; ^c^ cannot be calculated because >50% of patients were alive.
